# Tactile feedback is an effective instrument for the training of grasping with a prosthesis at low- and medium-force levels

**DOI:** 10.1007/s00221-017-4991-7

**Published:** 2017-05-26

**Authors:** Alessandro Marco De Nunzio, Strahinja Dosen, Sabrina Lemling, Marko Markovic, Meike Annika Schweisfurth, Nan Ge, Bernhard Graimann, Deborah Falla, Dario Farina

**Affiliations:** 1 Centre of Precision Rehabilitation for Spinal Pain (CPR Spine), School of Sport, Exercise and Rehabilitation Sciences, College of Life and Environmental Sciences, University of Birmingham, Edgbaston, Birmingham, B15 2TT UK; 2 Department of Translational Research and Knowledge Management, Otto Bock HealthCare GmbH, Max-Näder-Straße 15, 37115 Duderstadt, Germany; 3 Neurorehabilitation Systems Research Group, Clinics for Trauma, Orthopaedic and Plastic Surgery, University Medical Center Goettingen, Georg-August University, Von-Siebold-Str. 3, 37075 Goettingen, Germany; 4 Department of Bioengineering, Imperial College London, Royal School of Mines, London, UK

**Keywords:** Tactile stimulation, Visual feedback, Grasping force control, Prosthetic grasping, Myoelectric control, Feedforward control, Anticipatory mechanisms, Internal model

## Abstract

Grasping is a complex task routinely performed in an anticipatory (feedforward) manner, where sensory feedback is responsible for learning and updating the internal model of grasp dynamics. This study aims at evaluating whether providing a proportional tactile force feedback during the myoelectric control of a prosthesis facilitates learning a stable internal model of the prosthesis force control. Ten able-bodied subjects controlled a sensorized myoelectric prosthesis performing four blocks of consecutive grasps at three levels of target force (30, 50, and 70%), repeatedly closing the fully opened hand. In the first and third block, the subjects received tactile and visual feedback, respectively, while during the second and fourth block, the feedback was removed. The subjects also performed an additional block with no feedback 1 day after the training (Retest). The median and interquartile range of the generated forces was computed to assess the accuracy and precision of force control. The results demonstrated that the feedback was indeed an effective instrument for the training of prosthesis control. After the training, the subjects were still able to accurately generate the desired force for the low and medium target (30 and 50% of maximum force available in a prosthesis), despite the feedback being removed within the session and during the retest (low target force). However, the training was substantially less successful for high forces (70% of prosthesis maximum force), where subjects exhibited a substantial loss of accuracy as soon as the feedback was removed. The precision of control decreased with higher forces and it was consistent across conditions, determined by an intrinsic variability of repeated myoelectric grasping. This study demonstrated that the subject could rely on the tactile feedback to adjust the motor command to the prosthesis across trials. The subjects adjusted the mean level of muscle activation (accuracy), whereas the precision could not be modulated as it depends on the intrinsic myoelectric variability. They were also able to maintain the feedforward command even after the feedback was removed, demonstrating thereby a stable learning, but the retention depended on the level of the target force. This is an important insight into the role of feedback as an instrument for learning of anticipatory prosthesis force control.

## Introduction

Grasping is a complex action governed by a sophisticated coordination between several body systems (Georgopoulos and Grillner 1989; Marteniuk and Bertram 2001; van der Wel and Rosenbaum 2007). In daily life activities humans take advantage of sensorimotor integration in the control of grip force, learning through experience how to anticipate forces required to grasp an object (Flanagan et al. 2001; Johansson and Flanagan 2009). Previous studies have shown that grip force is tightly coupled to load force and that both grip and load force are modulated in an anticipatory manner as a function of the properties of the object (size, shape, and contact surface) (Johansson and Westling 1988; Johansson and Flanagan 2009; Hermsdorfer et al. 2011). To account for these anticipatory mechanisms, the concept of internal forward models that predicts the consequences of our movements has been proposed (Wolpert and Miall 1996; Wolpert et al. 2001). To predict grip forces, an efferent copy of the motor command is processed by an internal representation of the dynamics of the body segments involved in the motor task and the object to be grasped (Hermsdorfer et al. 2008). In addition, humans create and rely on the inverse models, which directly compute feedforward commands required to accomplish the task (e.g., estimating a force to grasp and lift a full bottle) (Hermsdorfer et al. 2011; Nowak et al. 2013). Here, the feedback is used to detect state transitions (e.g., contact and liftoff) triggering the release of appropriate motor programs and/or to online modulate the feedforward commands to correct errors due to uncertainty in modeling and/or environment (e.g., correcting the overshoot if the bottle was empty) (Flanagan and Wing 1997; Flanagan et al. 2001; Green et al. 2010). Somatosensory information plays a fundamental role in the learning, maintenance, and updating of these anticipatory mechanisms (Witney et al. 2004; Hermsdorfer et al. 2011; Jarrasse et al. 2013) and predictive force control requires at least intermittent cutaneous and proprioceptive feedback to signal the effectiveness of descending motor commands and to update internal models (Nowak et al. 2004).

In the case of an amputation of the hand, or a congenital absence of this body part, the grasping functions can be reestablished using a myoelectric prosthesis (Belter et al. 2013), although with a complete loss of cutaneous and proprioceptive sensory information. Despite the importance of sensory feedback in human motor control, none of the commercially available devices apart from a recently presented system (VINCENTevolution 2, Vincent Systems GmbH, DE) provide direct somatosensory feedback about the state of the prosthetic hand. The prosthetic users are, therefore, forced to compensate for the lack of direct feedback by attending to the alternative sources of information that are intrinsically available in the prosthesis (e.g., visual assessment, socket vibration, and motor sound). However, this requires a constant focus on the device and it can, therefore, be cognitively taxing. Provision of feedback is rated by the users as an important goal in prosthetic rehabilitation (Smurr et al. 2008; Bouwsema et al. 2014). Closing the loop might improve the consistency and efficacy in prosthesis control as well as facilitate the embodiment of the artificial system into the body scheme of the user, and thereby potentially increase the use and acceptance of the prosthesis (Peerdeman et al. 2011; Resnik et al. 2012).

Recent studies have extensively investigated methods to provide direct somatosensory feedback using closed-loop control and sensory substitution (Antfolk et al. 2013b). In this approach, the prosthesis is equipped with sensors, from which the data are acquired online and translated, with a coding scheme, into stimuli delivered to the user. The stimulation may be delivered to the peripheral nerves (Raspopovic et al. 2014), or to the brain (Tabot et al. 2013), or, non-invasively, over the skin using surface electrotactile or vibrotactile stimuli (Kaczmarek et al. 1991). In the latter case, one or more vibration motors are placed on the skin, and the feedback information is communicated by modulating the vibration intensity or frequency (parameter coding) (D’Alonzo et al. 2014b) or by changing the location of vibration delivery (spatial coding) when multiple vibrators are available (Saunders and Vijayakumar 2011; D’Alonzo et al. 2014a).

Most studies investigating feedback in prosthetics selected the grasping force as the variable to transmit to the user, since this variable cannot be easily determined by vision (at least for stiff objects) (Cipriani et al. 2008; Peerdeman et al. 2011; Antfolk et al. 2013a, b; Jorgovanovic et al. 2014; Dosen et al. 2015). Moreover, in amputees, grasping force feedback could partially substitute the lost cutaneous sensory information, which is fundamental to calibrate the accuracy of acquired internal models (Augurelle et al. 2003; Hermsdorfer et al. 2008). However, there is a lack of studies on the role of feedforward and feedback mechanisms in achieving a consistent anticipatory grasping force control of myoelectric closed-loop hand prosthesis. Previous studies aimed at demonstrating that prosthesis users employ internal models when reaching (Metzger et al. 2010), grasping, and moving objects (Weeks et al. 2000; Lum et al. 2014), with open-loop controlled prostheses (without tactile feedback); nonetheless, the accuracy of the reaching task as well as that of the force scaling and timing might have been supported by the sensory information coming from the residual limb (Macefield et al. 1996; Macefield and Johansson 1996; Nowak et al. 2002; Hermsdorfer et al. 2008). However, the accuracy of these models is poor and grip force modulation is significantly impaired. A recent study (Saunders and Vijayakumar 2011) demonstrated that feedback was beneficial only when a feedforward uncertainty was introduced in the control loop, while, when the prosthesis response was perfectly predictable and consistent, the provision of feedback failed to improve the performance. However, this result may be limited to the relatively simple task studied where the grasping prosthetic hand was controlled in a binary way via force threshold switching using a fingertip flexion or extension. Another study (Dosen et al. 2015) specifically investigated the effect of uncertainty in control (joystick vs. myoelectric control) and system (ideal vs. real prosthesis) on the learning and maintenance of feedforward internal models during repeated grasps supported by an explicit force feedback. The results demonstrated that the subjects could utilize the feedback to learn feedforward control of the prosthesis grasping force. However, the control performance deteriorated fast in the absence of feedback, especially when using the myoelectric command interface. However, the control loop in that study was closed using a visual interface (a bar on a computer screen), which does not correspond to realistic conditions, and the test was done for only one force level. Investigating feedback in prosthetics using a setup similar to that of Dosen et al. (2015) with tactile feedback interface and multiple force levels would provide important novel insights into the practical effect of feedback on grasp control. Therefore, the main aim of this study was to investigate how prosthetic grasping force control improves, in terms of accuracy and precision, using a tactile feedback interface. A sequence of fast grasping trials has been selected to explore prosthetic control, mimicking the manner grasping is routinely executed in daily life. The subjects were asked to apply anticipatory grasping force control and use the feedback information (the generated grasping force) to learn, update, and maintain the feedforward (anticipatory) command. As explained by Hermsdorfer et al. (2011), able-bodied subjects estimate the force that is required to grasp an object based on the object properties. Similar mechanism has been observed in amputee subjects controlling open-loop prosthesis (Lum et al. 2014). In both cases, the force anticipation is translated in feedforward commands executing the grasp. The second aim of the study focuses, therefore, on exploring the ability of learning, updating, and maintaining an internal model of a myoelectric prosthesis to accurately and precisely control its grasping force in a feedforward manner.

## Methods

### Experimental setup: closed-loop system

**Fig. 1:**
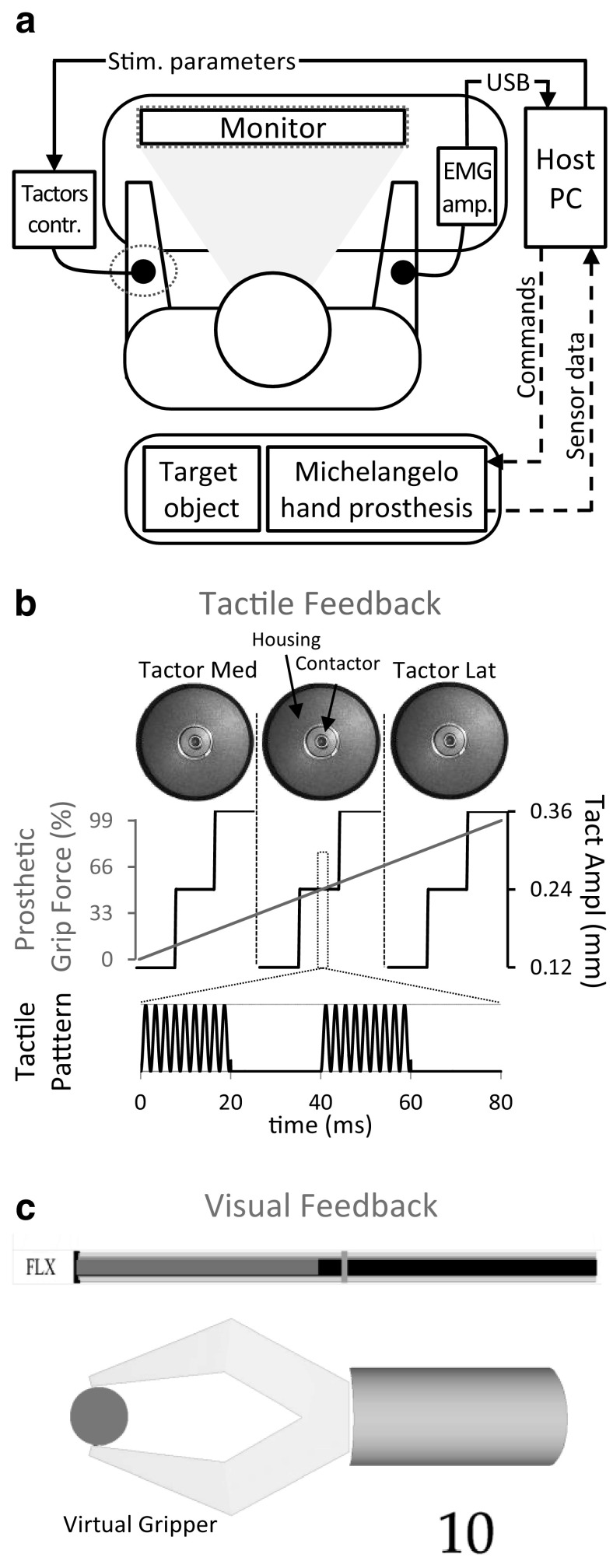
Experimental setup (**a**) and systems to deliver Tactile (**b**) and Visual (**c**) Feedback. A simulated model of a prosthesis (*Virtual Gripper*) and a real prosthesis (*Michelangelo hand prosthesis*) were controlled proportionally using EMG activity from the wrist flexor muscle. The subject was seated at the desk in front of a monitor always showing the prosthesis aperture through the Virtual Gripper. The Tactile Feedback (**b**): an array of 3 C2 tactors (EAI, USA) was used to feedback 9 levels of grasping forces. Bursts of 230 Hz delivered at 30 Hz were used as vibrotactile stimulus, and 3 amplitudes of vibration (min, med, max) for each tactor coded the 9 force levels. The Visual Feedback (**c**) was realized with a moving red bar showing the actual grasping force level of the prosthesis. A *green vertical line* represented the Target Force Level to be achieved for that run. An electromyography amplifier and the tactor controller were both connected via USB to the host PC. The prosthetic hand and the target for grasping were placed behind the subject (color figure online)

The experimental setup is shown in Fig. 1a. A dry double differential electrode for surface EMG detection (Myobock, Ottobock Healthcare GmbH, AT) was used as a command interface. The surface EMG electrode was placed on the volar side of the forearm (Fig. 1a), over wrist and hand flexor muscles and the acquired and processed EMG signal was used to proportionally control a prosthetic hand (Michelangelo Hand, Otto Bock Healthcare GmbH, AT). To avoid indirect feedback information induced by the movement of the prosthesis, the prosthetic hand was detached and placed behind the subject who wore ear plugs, blocking thereby both visual and auditory information. The hand was fixed using a vice and a rigid object was positioned in between the prosthetic fingers so that the prosthesis grasped the object when closing. To ensure standardized conditions across the sessions, the subject looked at a computer monitor placed on a table in front of him at approximately 50 cm, showing an animated graphical representation of the prosthesis (Fig. 1c) and of the rigid object to be grasped (Fig. 1c, green circle). During all the sessions, the gripper provided visual feedback of the prosthesis motion since the aperture of the gripper was updated online by reading the sensor data from the prosthesis (see below). This experimental design has been selected to obtain a consistent setup across the subjects and conditions as well as to specifically investigate the effect of a single feedback source (tactile interface). The fast sequence of continuous grasping trials indeed simulates a routine grasping action in daily life (e.g., grasping a glass to drink). However, in the real-life use, the prosthesis will be attached to the subject and indirect feedback sources such as motor sound and vibration through the socket will be available.

The hand grasped the same object across trials, starting from the same position (fully open). Therefore, the amount of movement until contact was identical across trials, from the full aperture to the object contact. The hand closing velocity, however, depended on the subject command generated via muscular activity (EMG signal proportionally controlling the Michelangelo hand). In general, to reach higher target forces (high vs. medium vs. low), the subjects produced stronger contractions and, therefore, the prosthesis closed faster.

The Michelangelo hand is equipped with position and force sensors. The position sensor measured the hand aperture on 100 discrete levels from fully open (~11 cm aperture) to fully closed hand. The force sensor placed between the index finger and the thumb measured the grasping force on 90 levels, up to a maximum force of ~100 N. Since the virtual object and the gripper were modeled as rigid, watching them did not allow the extraction of any information on the generated grasping force. The virtual setup provided a standardized feedback of the prosthesis motion to all the subjects, eliminating potential confounding factors (e.g., different viewing angle due to changes in subject height).

The online control loop was implemented using a flexible framework for the assessment of the human manual control (Dosen et al. 2014) developed in MATLAB Simulink 2013 using the Real-time Windows Target toolbox (MathWorks, US) and executed at the sampling frequency of 100 Hz. The host PC (Fig. 1a) was a standard desktop computer running the closed-loop system and communicating with the prosthesis via a Bluetooth. The active electrode sampled the EMG at 1 kHz and computed its root mean square (RMS) value from 250-ms data intervals, with 80% of overlap. The prosthesis controller (AxonMaster) sampled the sensor data, including position, force, and processed EMG at 100 Hz and sent them to the host. The host PC received the processed EMG signal and used it to proportionally control the velocity of the prosthesis during closing and grasping force (after contact). The myoelectric control was calibrated so that approximately 80% of the maximum voluntary contraction (MVC) resulted in the maximum command. The prosthesis commands were calibrated so that *X*% of muscle activation led to *X*% of grasping force, where *X* is the number between 0 and 100% indicating percent of MVC and maximum prosthesis force, respectively. The release and opening of the hand were automatically triggered when the command signal returned from a positive value back to zero. The hand opened completely, and the fully open hand was the starting position in each grasping trial. The position and force sensor data were used to update the aperture of the virtual gripper and provide the visual and vibrotactile feedback of the grasping force, respectively (see next section). The total time to operate the myoelectric control, including the data transfer from the amplifier to the host PC and then to the prosthesis, resulted in a delay of ~60 ms (Dosen et al. 2015). In preliminary tests, we noticed that the delays in the control loop did not influence the performance because the grasping force control was conducted in a feedforward manner.

### Experimental setup: visual and vibrotactile feedback

In some conditions (see experimental protocol), visual or vibrotactile feedback of grasping force was provided to the subject. The visual feedback was selected as an interface with a high resolution and easy interpretation (Ninu et al. 2014; Dosen et al. 2015), and tactile feedback was chosen as a practical and commonly used solution for sensory substitution in prosthetics (Schofield et al. 2014; Cordella et al. 2016). The role of the visual feedback condition was to test if the feedback of higher fidelity can contribute to better performance compared to the practical tactile interface. The visual feedback (Fig. 1c) was implemented using a horizontal red bar indicating the actual grasping force applied by the prosthesis on the target object. The length of the bar was proportional to the measured grasping force. A green vertical line marked the reference force for the given trial (Target Force Level). The vibrotactile feedback was provided using a set of vibration motors placed around the forearm (Fig. 1b).

An array of three vibrators (C-2 tactors, ATAC Technology, Engineering Acoustics Inc., US) was placed around the non-dominant arm, 5 cm distal to the elbow joint. Tactor 1 was the most medial and tactors 2 and 3 were more lateral and the covered part of the extensor muscles of the forearm, with more than 4 cm of distance between subsequent tactor contactor heads. The distance between the first and the last tactor covered almost 50% of the circumference of the subject’s arm, to assure good spatial discriminability of the tactor sensations (Jones and Safter 2008). The tactors were driven by a tactor controller connected to the host PC via USB port. The vibration parameters (intensity and frequency) were adjusted online by sending simple text commands.

To provide feedback, the tactors delivered vibration bursts of 230 Hz, at a frequency of 25 Hz. Each high-frequency burst lasted 20 ms with 20-ms break inbetween the bursts. Each tactor coded three grasping force levels through the intensity of its vibration (Fig. 1b). The selected intensity levels corresponded to 0.12, 0.24, and 0.36 mm of contactor oscillation. Therefore, the subjects could overall distinguish nine levels of tactile stimulation. The total force range (100%) of the prosthesis was divided into nine equal subranges (11% each), which were then associated with the nine tactile stimuli (3 tactors × 3 intensities). The feedback occurred as soon as the prosthesis hand closed (force >0) and remained active until the prosthesis opened. The selected tactile stimulation setup allowed for the high discriminability of the tactile stimulation levels (84% of correct stimulus identification during familiarization training, see Experimental Protocol for further details) and a good exploitation of tactile sensory ability without using a cumbersome tactile display (Jones and Safter 2008).

### Experimental task

The task for the subjects was to repeatedly grasp the target object by applying the instructed level of grasping force. In each trial, the subject closed the prosthesis starting with the hand fully opened (initial configuration) and performed the grasp using a continuous contraction of the wrist flexor muscles. After the grasp, the subjects were instructed to relax the muscles, which triggered the automatic opening of the hand back to initial configuration. The subjects were instructed to close the prosthesis in one fast and direct attempt without steering the force after contact, mimicking the routine execution of grasping action in daily life. To achieve this with prosthesis, the subjects increased the muscle activation to a desired level and maintained the contraction until the prosthesis closed and grasped the object. The subjects performed this task in different feedback conditions and target force levels, as explained in the next section. The higher the desired level of force, the stronger the muscle contraction that the subjects were required to produce and the faster the prosthesis closed. The subjects were, therefore, required to produce the desired muscle activation level faster for higher forces. However, there was no hard time limit in the control system that would disable further input into the prosthesis after contact. The grasping was performed fast and the subjects anticipated the requested amount of force to be generated (feedforward control), as the force feedback was received after the prosthesis hand closed. Therefore, the feedback information was not used to modulate the force during an ongoing trial. However, the assumption was that the feedback could still be used to adjust the motor command across trials, thereby learning the feedforward model and improving the performance. The feedback provided the information on the force generated in the current trial, and based on this, the subject could decide if he/she needed to increase or decrease the muscle activation in the next trial.

### Experimental protocol

The tests were performed in ten able-bodied subjects (age 24.2 ± 4.6 years, mean ± SD) who signed an informed consent form for the experimental protocol that was approved by the local ethics committee.

The subjects were seated comfortably in a chair in front of a desk, and they used their dominant hand to control the prosthesis. The skin of the subject was prepared with a small amount of abrasive paste (EVERI160SPE, Spes Medica S.r.l., Italy) and the Myobock electrode was placed on the central part of the belly of the hand and wrist flexor muscles of the dominant side. The tactors were then fixated by an adjustable, textile strap. The positions of the electrode and the tactors were marked on the skin with a pen to ensure consistent placement during the second session. The dominant forearm was placed into an orthopedic splint to allow prosthesis control using isometric contractions.

**Fig. 2:**
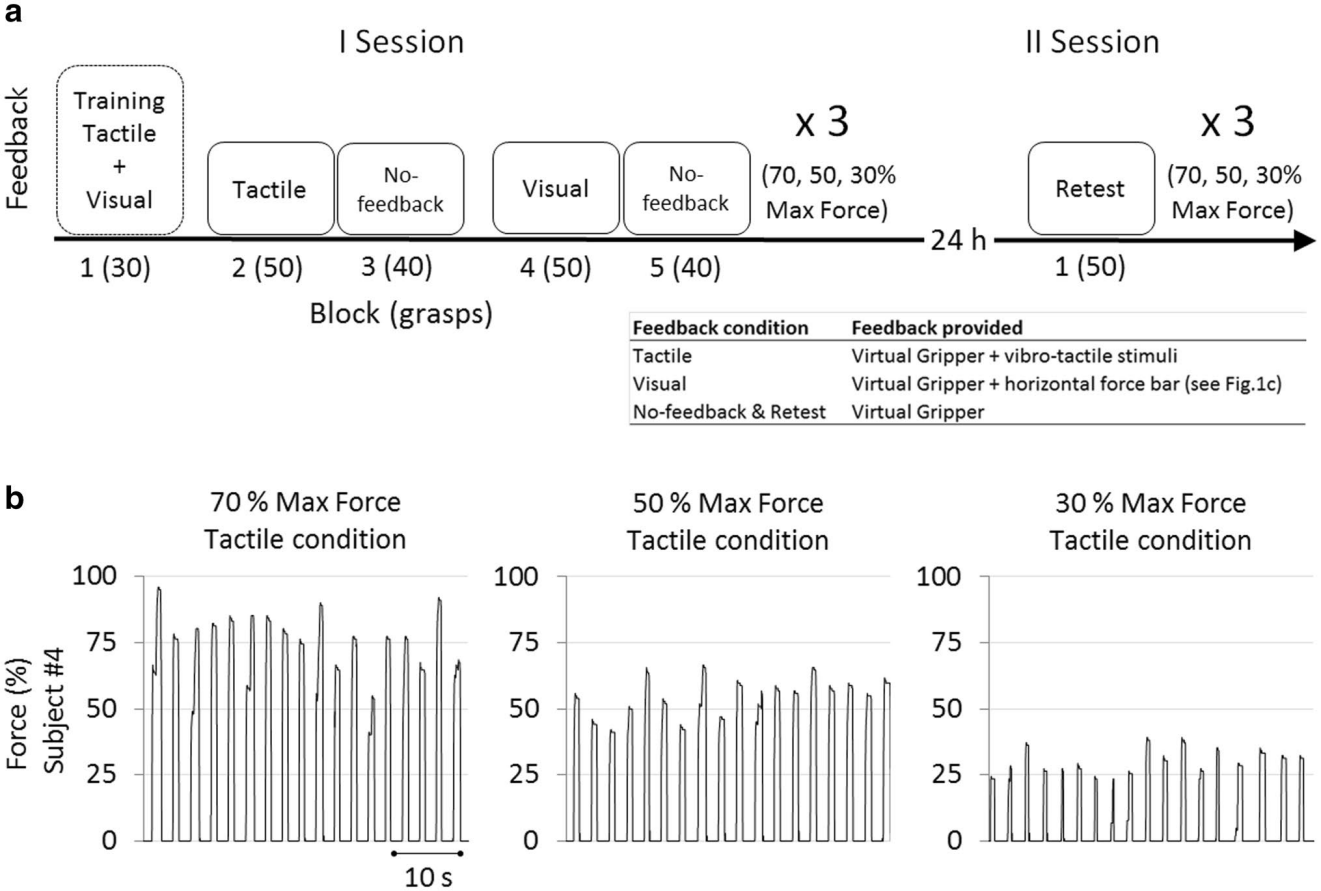
**a** Experimental protocol: the first session of the protocol contains *5 blocks*. In each *block* the feedback type is reported (e.g., first training *block* is executed providing both vibrotactile and visual feedback). The number of trials (*grasps*) executed in each *block* is reported in *brackets*. The second session, realized almost 24 h later contains just one *block* of 50 trials. First and second sessions are repeated three times, one for each target force level (70, 50, and 30% of the prosthesis maximum grasping force). “Retest” and “No-feedback” conditions contain the same feedback type (just Virtual Gripper, see Fig. 1, without the *horizontal* force feedback *bar*). **b** Part of *block 2*: prosthetic force (% of maximum force generated by the prosthesis) exerted by one subject grasping trials, during the three different target forces (70, 50, and 30%) for one feedback condition (*Tactile*)

Each subject was tested in two sessions (Fig. 2a) on consecutive days. The first session consisted of three repetitions of a series of five blocks, comprising 30, 50, 40, 50, and 40 grasping trials, respectively. Before starting with the data acquisition, the principle of functioning of the prosthesis, including the proportionality between the EMG, closing velocity and grasping, as well as the operation of the tactile feedback interface was explained to the subjects both verbally and with practical demonstrations until the subjects was confident in these operations. Afterwards vibration codes were presented randomly to the subject and he/she was asked to identify and report the transmitted force level. This task was repeated 50 times, after which the subjects could correctly identify the stimulation levels in 83.56 ± 8.6% (mean ± standard deviation) of the attempts.

The subjects then performed a first block of trials, devoted to general training, during which the subjects received simultaneous tactile and visual force feedback. The aim was that the subjects learn a general principle of prosthesis operation (e.g., proportionality between contraction, velocity and force) and feedback interpretation (i.e., mapping between tactile sensation and force level). The subjects were instructed to attain a target grasping force changing randomly between 20 and 80% of the maximum prosthesis force every two grasping trials.

The data acquisition started with the second block (tactile feedback). During blocks 2 and 4, the subjects were provided with tactile feedback only and visual force feedback only, respectively (reported as “Tactile” and “Visual” condition in the Figures). This was a focused training for generating the same level of target force across the trials. Just before the grasping trials in block 2 started (“Tactile”), the vibration code indicating the target force that needs to be achieved was delivered to the subject for a few seconds, so that the subject was reminded about the tactile sensation that will be produced in the case of the correctly generated target force level. In block 4 (visual force feedback), the target force was indicated as a green vertical line within the red bar depicting the measured grasping force. During blocks 3 and 5, the subjects received no force feedback (reported as “No-feedback” condition). The subjects were instructed to aim for the same target force as in the other trials of the current run (Fig. 1c). The blocks 2–5 were repeated three times with a different target force in each run, i.e., 70, 50, and 30% of MVC for runs 1–3, respectively. The first session lasted approximately 1.5 h.

The blocks with feedback assessed the quality of closed-loop force control using different sensory modalities, i.e., practical tactile and ideal visual interface. These blocks also served as training during which the subject relied on the feedback to tune the feedforward commands through trial by trial learning, possibly facilitating the learning of a stable feedforward internal model. Each feedback condition (tactile and visual) was followed by a block of grasping trials with the same target force but with the feedback removed (No-feedback), with the aim of assessing the retention of the learned feedforward models.

The second session consisted of the three runs as well, but this time each run included only one block. The block comprised 50 trials and no feedback was provided to the subjects. The aim was to retest the learned performance in open-loop (feedforward) grasping force control (Fig. 2a “Retest” block), assessing the retention 1 day after the training. The second session lasted approximately 30 min as all subjects were already familiar with the system operation, tactile feedback, and the experimental protocol.

In both sessions, the subjects performed the trials at a comfortable pace, usually with few seconds between trials. One- and five-minute breaks were included between blocks and between the three runs for the 3 target forces, respectively.

### Data analysis

The maximum prosthesis force reached during a grasping trial was adopted as the trial outcome, hereafter named the generated force. The peak of prosthetic grasping force has been used as outcome parameter taking into account the type of the executed task and the characteristic shape of the force developed during the trials (Fig. 2b). To initiate the grasp, the subjects activated the flexor muscle to the desired level, and the prosthesis closed and produced the corresponding force. The subjects then relaxed the muscle while the prosthesis maintained the force (non-backdrivable operation) and then opened automatically. The profile of the generated force, therefore, exhibited a plateau which corresponded to the signal maximum. Figure 2b reports the characteristic peaked shape of the prosthetic grasping force developed at each trial (single peak) during the three different target forces for just one subject and one feedback condition (tactile). Median and interquartile range (IQR), of generated forces during the last 40 trials of each block, were extracted for each subject and pooled for each session (2), feedback condition (5: tactile, No-feedback, visual, No-feedback, Retest), and target grasping force level (3: 70, 50, 30%). The dispersion of the medians (medians’ variance) of the generated forces was used to evaluate the overall accuracy of the grasping force control for the subject group, while IQR assessed the precision.

### Statistical analysis

The Kolmogorov–Smirnov test was executed to assess the normality, and the test showed that the data (Median and IQR) were normally distributed. To test the consistency in performance (accuracy) across subjects, Bartlett’s test has been used to compare the equality of variance of the median values between conditions. Similar dispersion of the medians in the two conditions (e.g., feedback and no-feedback) indicated that the overall performance remained similarly consistent. If the dispersion substantially increased, this was an indication that some subjects exhibited a loss of performance (accuracy). Paired Student’s T test of IQR values has been used to assess the grasping force precision across conditions. Bonferroni correction has been used for both of the tests. The value of *p* ≤ 0.05 was considered as statistically significant. All computations were performed using the software package IBM SPSS Statistics Version 22.

## Results

**Fig. 3:**
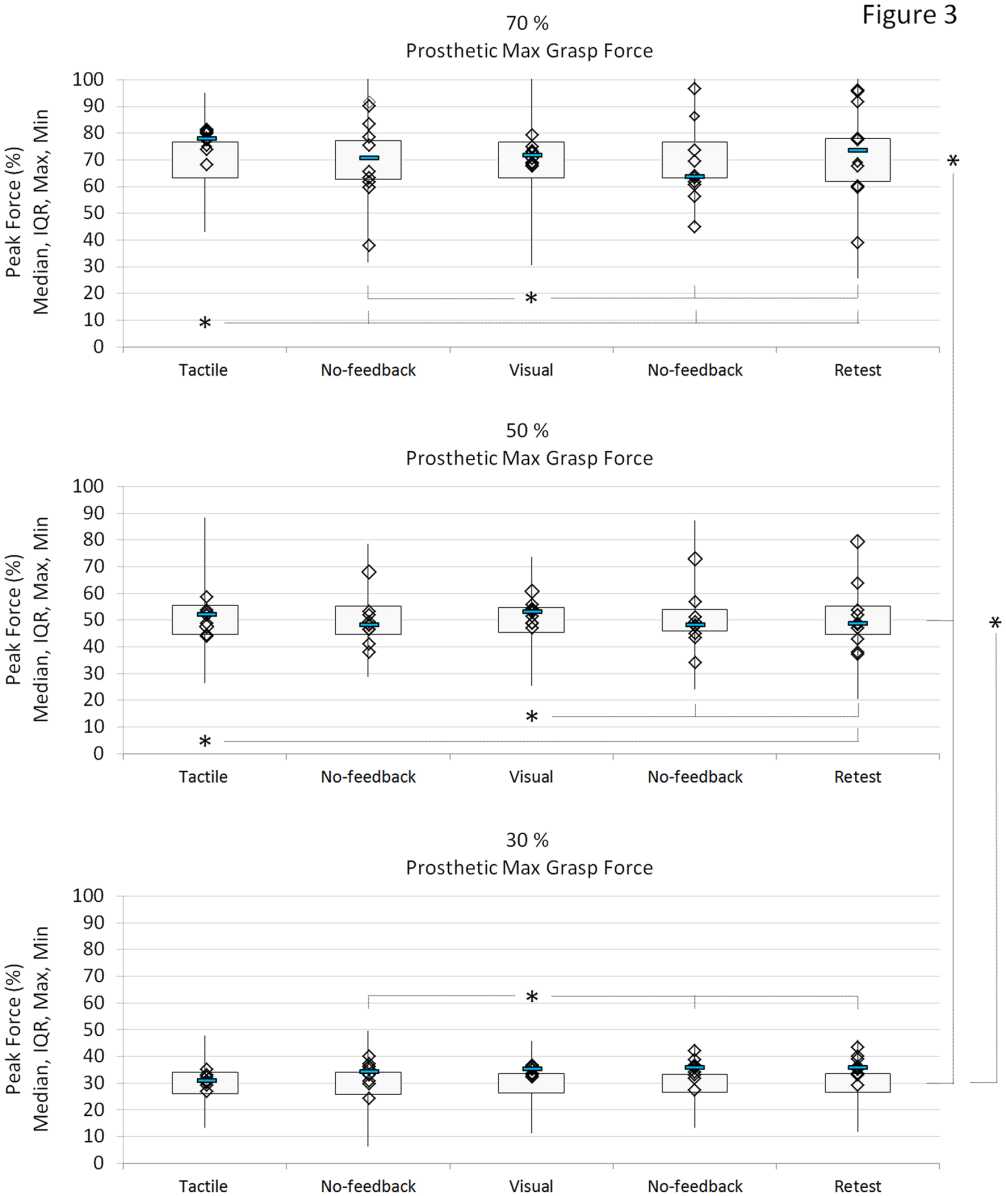
Median values for each subject (*diamonds*) and the overall median (*blue rectangle*) superimposed on *box plots* reporting the group averaged IQR (Maximum and Minimum force peak values as *whisker*s) under the three target force levels, 70, 50, and 30% (from *top to bottom*), for each of the feedback condition (*Tactile*, *No-feedback*, *Visual*, *No-feedback*, and *Retest*). The data report the average prosthetic force accuracy (statistics across feedback conditions) and precision (statistics across target force level). Note that the *box* for the mean IQR is centered at the target force so that the precision can be easily compared across condition. The *asterisks* and *horizontal bars* represent significant differences (*p* < 0.05) between the condition labeled with the *asterisk* and the conditions pointed by the *bars* (color figure online)

Figure 3 reports the accuracy and precision in grasping force control across the tested conditions. The medians of the reached force levels, for each subject, are displayed as markers and the group mean IQR (average across subjects) is represented as a box, centered on the value of the target force (e.g., 70, 50 or 30%). The vertical lines depict the overall maximum and minimum of the generated forces. The statistically significant differences for the median dispersion are indicated as horizontal bars inside each plot and as vertical bars outside the plots (on the right side) for the IQR.

The tactile and visual feedback conditions across all the target forces were characterized with a good accuracy. The medians of all subjects were concentrated closely around the target force. The performance was consistent across subjects and all the subjects were similarly accurate in force control. The average absolute deviation of the medians from the target for the visual/tactile feedback was 4.7/1.8%, 3.7/3.6%, and 3.2/7.6% at the force of 30, 50, and 70%, respectively. There was no statistically significant difference in the dispersion of the medians between the two feedback modalities for the same target force and across them. Hence, the visual feedback did not significantly improve the performance with respect to the tactile modality.

When the feedback was removed, the medians in general became more dispersed. This, however, depended on the target force and it was also subject specific. Some subjects remained accurate even at the highest force (e.g., 0.4% lower than the target), while some shot significantly off the target (e.g., 21.6% higher than the target). This seems to be a consistent trend; the subject who did not retain the accuracy from tactile to No-feedback condition also failed to retain the performance from visual to the following No-feedback condition (e.g., in one subject, a deviation from the target force of 9.9 and −31.7% passing from tactile to No-feedback, −2.3 and −24.9% from visual to No-feedback).

Importantly, at 30%, the performance was consistent across subjects and conditions. Namely, the dispersion of the medians remained similar when the tactile feedback was removed, i.e., no statistically significant difference between the tactile and following No-feedback condition and this also held for the retest on the day after. Therefore, the medians of all the subjects were consistently concentrated around the target force and the subjects were accurate [±3.5% standard deviation (SD)] irrespective of the feedback and session. At 50%, the medians remained concentrated when the tactile feedback was removed, indicating consistent accuracy across subjects, but the medians spread significantly more during the retest (*p* = 0.005). Without feedback, in most of the subjects the medians were still close to the target (±6.9% SD), although there were subjects who were more off (e.g., 18.1% higher than the target force at No-feedback following tactile condition) especially during the retest (e.g., 29.4% higher than the target force). Finally, at the highest target force (70%), the medians dispersed substantially as soon as the feedback was removed, i.e., the dispersion in No-feedback and retest was significantly larger compared to that in tactile condition (*p* < 0.001). Therefore, once the feedback was removed, the subjects exhibited a substantial loss of accuracy, and they were significantly off (±15.5% SD) with respect to the target.

Across target forces, there was no difference in median dispersion between 30 and 50% for non-feedback conditions and retest, while the dispersion increased at 70%. The performance across subjects was more consistent in the visual feedback condition compared to conditions with no feedback (*p* < 0.015).

The precision exhibited a different trend from the accuracy. There was no statistically significant difference comparing the IQR values across the conditions (from tactile to retest condition) for each of the three target force levels. Therefore, the presence or absence of feedback did not affect the grasping precision. However, the average IQR (Fig. 3) at 70% target force was statistically larger than that at 50 and 30%. Similarly, the average IQR at 50% was significantly larger than the IQR at 30% target force (*p* < 0.01).

The IQR values for the three target force conditions (on average 14.2, 9.8, and 7.5% for 70, 50, and 30% of target force, respectively) showed a very strong linear correlation (*p* < 0.001) with the target force value (Pearson product-moment correlation coefficient, *r* = 0.82). The precision of prosthetic grasping force control seems almost independent from the availability of sensory feedback while it changes accordingly to the grasping force intensity.

## Discussion

The main aim of the present study was to investigate the ability of naïve subjects to improve feedforward prosthetic grasping force control using a tactile feedback interface. The hypothesis was that the closed-loop control of prosthetic grasping force could facilitate the acquisition, updating, and possibly retention of an internal model of feedforward control during myoelectric prosthesis grasping.

The results indeed demonstrated that the provision of tactile feedback led to accurate control (Fig. 3, visual and tactile conditions). With the feedback, the subjects were consistently accurate at all force levels. On the other hand, the precision decreased with an increase in the force level. Therefore, even when the feedback was not directly used to modulate the force during an ongoing trial, it could be used as an effective instrument for adjusting the feedforward motor command across trials. More specifically, the feedback was effective in adjusting the accuracy of grasping force control, whereas the precision was not affected by the presence of feedback. Importantly, there was no significant difference in performance (accuracy and precision) across target force levels when using vibrotactile compared to visual interface. This is an encouraging result for the prospect of implementation of closed-loop prosthesis, demonstrating that a practical vibrotactile interface, although limited in resolution, can lead to a prosthesis control which is close to the benchmark level (continuous visual information). The similar result was reported by Dosen et al. (2016) where the electrotactile feedback using multichannel electrode led to a similar performance as the visual feedback. The interface in this study combined spatial and intensity coding to obtain nine stimulation codes. The codes were, therefore, easy to discriminate providing thereby the feedback that was reliable albeit with the limited resolution. The surface myoelectric control is noisy and variable and this is a likely reason why feedback of a much higher resolution (visual) at the end failed to significantly improve the performance (accuracy as well as precision). The subjects could not exploit the finer information available due to an inconsistent command interface. Several previous studies have emphasized the impact of the consistency and reliability of the command on the overall closed-loop performance (Antfolk et al. 2013b; Ninu et al. 2014; Dosen et al. 2015).

A second conclusion is that training grasping with force feedback allowed the subjects to develop an internal model of the feedforward prosthesis control. The effectiveness of feedback in training the subjects to acquire this model depended on the target force level. In general, some subjects maintained the accuracy when the feedback was removed within the session and even across sessions (Retest). However, as the target force increased, more subjects exhibited a substantial loss of accuracy when they were deprived of explicit feedback. Therefore, the subjects not only adjusted the motor command to a proper level using feedback, but they could also reproduce this command when the feedback was removed, indicating a stable learning. However, as the target force increased, the ability to retain the feedforward command in the absence of feedback gradually decreased. At 50%, the performance remained consistent across subjects within the session and the dispersion of the medians increased only during the retest. At 70%, however, the medians spread substantially as soon as the feedback was removed (hence within the session).

The precision exhibited similar trend across the force levels, i.e., an increase at the higher forces, but the precision was surprisingly consistent across the conditions. This provides an insight into the process of motor adaptation facilitated through feedback. During consecutive grasping, the subjects repeatedly produced the muscle activation level that they estimated would result in the desired grasping force. The vibrotactile feedback provided information on the actually generated force, which the subjects used to modulate the contraction in the next grasp. Across trials, therefore, the subjects adjusted their motor command at the proper level (steady state, Fig. 2b). If this level was successfully memorized (as was the case for medium and low levels), the subjects were able to generate the forces accurately even after the force feedback was removed. However, the process of generating repeated contractions as well as recorded myoelectric signals are characterized with an intrinsic variability (see next section), which cannot be decreased even when the feedback on the generated force is provided. Therefore, even if the mean level of muscle activation was properly remembered by the subject, the overall performance of force control was limited by the dispersion of the forces as determined by the intrinsic precision. In summary, when asked to generate a certain force repeatedly, the subject aims at reproducing the learned contraction level, which becomes translated into prosthesis force with a precision that depends on the contraction magnitude (higher force, higher variability).

The present study complements and extends the previous work by Dosen and colleague (Dosen et al. 2015) demonstrating that stable models can be acquired and retained even when using a noisy command interface (myoelectric control), provided that the force levels are low to medium and that the feedback is transmitted using a tactile interface.

The present study provides further insight into the operation and role of feedforward and feedback processes during closed-loop prosthesis control. More specifically, we investigated the ability of learning, updating, and maintaining an accurate internal model of a myoelectric prosthesis to consistently control its grasping force in a feedforward manner. In contrast to the previous work (Dosen et al. 2015), the present experiment evaluated the tactile feedback based on vibration and mixed coding, which is a practically relevant interface [instead of visual feedback used in (Jarrasse et al. 2013)]. Furthermore, the training of feedforward control was performed on multiple force levels (low, medium, and high) and the retention was tested within session as well as after 1 day.

In the present study, the learning was investigated in the context of repeated grasping of a single force level at a time. This corresponds to repeatedly grasping the same target object. The future work will consider a more realistic paradigm in which the subjects will need to produce randomly changing forces, as when grasping several objects of different properties (from delicate to robust).

Contrary to the continuous visual feedback, the tactile feedback was discretized in 9 levels. Nevertheless, the subjects could still modulate the prosthesis force in a continuous manner in both cases. This was outside the scope of the present study, but it is certainly of interest to investigate how the resolution of the feedback interface (fewer vs. more discrete levels) affects the closed-loop performance.

In the “No-feedback” conditions, the subjects did not receive an explicit feedback on force, but they could still observe the prosthesis motion. Therefore, they could estimate the velocity of closing and use this variable to maintain the desired grasping force across trials, as demonstrated by Ninu et al. (2014). More specifically, they could try adjusting the prosthesis velocity to that observed during the training. However, to which extent the subjects relied on this information cannot be discerned by the present study. This is an important question for the future work. In any case, the subjects will indeed have access to visual feedback during the real-life use of the prosthesis. In addition, vision will be supplemented by other sources of indirect feedback, such as motor sound and vibrations transmitted through the socket.

## Discussion of the results from a neurophysiological point of view

In this study the motor task was to control the muscular activation intensity of the wrist flexor to close the prosthetic hand and develop a target grasping force level. The grasping force normalized to prosthesis maximum was linearly proportional to the muscular activity normalized to MVC (calibrated prosthesis). Therefore, to accomplish the routine prosthetic grasp the subject was involved in finely tuning the amount of EMG activity to routinely reach a certain level of muscle contraction with a good accuracy and precision.

Moreover, a greater activation of the muscles results in higher neuromuscular noise levels which produce larger variability (Faisal et al. 2008). Over the normal force range of movement, the total noise affecting the activation of each muscle is not constant, but increases approximately linearly with the amplitude of the motor command signal (Meyer et al. 1988). This signal-dependent noise results directly from the physiology of the motor pool, in which motor units are recruited in order of increasing twitch amplitude (Jones et al. 2002).

The increased muscular activity, showed for higher target force, led to an increased signal variability as motor units were progressively recruited according to the size principle (higher number and larger size) (Henneman et al. 1965; Enoka and Fuglevand 2001). The linear dependency between neuromuscular noise and muscular activation level might explain the linear dependence between force precision of prosthetic grasping control (IQR) and the level of tested target force.

Moreover, these premises might account for the significantly noticeable decrease in the prosthetic grasping force accuracy under open-loop control (No-feedback and Retest) compared to closed-loop control, with both tactile and visual feedback, at the high target force level (70%). For high target forces, the subject was requested to execute a more demanding task using a control characterized by higher uncertainty (Franklin and Wolpert 2011) implying stronger focus on the feedback information (Laine et al. 2014). This is also in accordance with studies in the literature (Wei and Kording 2010; Johnson et al. 2014) demonstrating that the adaptation rate, which corresponds to the amplitude of feedback-driven corrections, increases with the certainty of feedback and uncertainty of feedforward control.

## Implications and conclusions

In most of the studies evaluating the application of tactile stimulation for closed-loop control in prosthetics, the feedback has been regarded as an add-on that should instantly improve the performance of the assistive device. In the present study, however, we investigated the potential of the feedback to operate as an instrument for trial to trial learning. This learning can lead to acquisition of a stable feedforward control scheme that can be used to operate the prosthesis at the same level of performance, even after the feedback has been removed. Importantly, the present study demonstrated that the learning is more effective when the forces are low or medium. This conclusion has practical implications. The feedback might not be required continuously while using the prosthesis. We can speculate that the benefits of feedback would be best expressed initially, in naïve users, when they are still learning how to utilize the prosthesis. Once the system is learnt, the feedback might be turned off or provided intermittently, i.e., from time to time to refresh (recalibrate) the feedforward model.
